# Papillary Thyroid Cancer With Pituitary Gland Metastasis: A Unique Encounter

**DOI:** 10.7759/cureus.38210

**Published:** 2023-04-27

**Authors:** Muhammad I Butt, Ahmad M Siddiqi, Faisal M Joueidi

**Affiliations:** 1 College of Medicine, Alfaisal University, Riyadh, SAU; 2 Medicine, King Faisal Specialist Hospital and Research Centre, Riyadh, SAU

**Keywords:** radioiodine treatment, panhypopituitarism, diabetes insipidus, pituitary metastasis, papillary thyroid cancer

## Abstract

The pituitary gland is a rare metastatic site, and thyroid cancer (TC) metastasis to the pituitary gland is immensely uncommon. We report the case of a 45-year-old male in whom pituitary metastasis (PM) discovery during the immediate postoperative period complicated the management of papillary thyroid cancer (PTC). His postoperative magnetic resonance imaging (MRI) of the pituitary lesion showed a progression in size with persistent optic nerve compression. The critical location of the pituitary lesion and the rapid progression dictated the treatment course. The pituitary lesion was non-iodine avid, and thus we opted for external beam radiation therapy (EBRT). He received 1,200 centigray (cGy) with Gamma knife radiosurgery with steroid cover. In our case, the aggressive histological and clinical variant of PTC consisted of multiple metastatic sites involving large volume pulmonary, skeletal, and chest wall lesions coupled with crucial macro metastatic pituitary mass. The patient was offered radioactive iodine to treat other iodine avid metastases in the lungs and bones and was also offered EBRT to target skeletal lesions. Systemic treatment with tyrosine kinase inhibitor was also discussed with the patient. Our case encourages clinicians to exercise vigilance and a high index of suspicion for PM when a patient with any pre-existing cancer presents with visual disturbance, cranial nerve deficit, or symptoms suggestive of hormonal deficiency. It also highlights the importance of involving endocrinologists before performing any surgery on the endocrine organs to ascertain the integrity of the endocrine function of the glands.

## Introduction

The pituitary gland is a rare metastatic site, representing only 1% of all patients operated on sellar or para-sellar lesions [1]. The posterior lobe of the pituitary gland is involved in approximately 85% of the cases, making central diabetes insipidus (DI) the most common presentation. The anterior lobe alone is involved in a minority of the cases affecting nearly 15 % of the patients [1,2]. This predilection for the metastasis into the posterior pituitary could be related to the direct blood supply it receives from the hypophyseal arteries and its large contact area with the adjacent dura [3].

Pituitary metastasis (PM) often originates from primary lung and breast cancers. Thyroid cancer (TC) accounts for 2% of all PMs [1]. However, metastasis to the pituitary gland is immensely uncommon. TC complicated by PM carries a poor prognosis and constitutes a diagnostic and therapeutic challenge.

We report the case of a 45-year-old male in whom the discovery of PM during the immediate postoperative period complicated the management of papillary thyroid cancer (PTC).

## Case presentation

A 45-year-old male presented to the surgical clinic with a one-month history of neck swelling, weight loss, palpitations, and loss of appetite. He was healthy before this presentation and had no head and neck radiation exposure history. He did not suffer from COVID-19 infection. There was no history of TC within the family. A physical examination of the neck revealed a hugely enlarged right lobe of the thyroid gland.

Biochemical workup showed a low free thyroxine (FT4) level of 10.4 pmol/L with an inappropriately low thyroid-stimulating hormone (TSH) level of 0.008 mU/L. The neck ultrasound (US) scan confirmed a suspicious nodule measuring 6 x 4 cm on the right lobe of the thyroid gland, along with multiple enlarged lymph nodes in the neck. The nodule's fine needle aspiration cytology was suspicious of malignancy.

**Figure 1:**
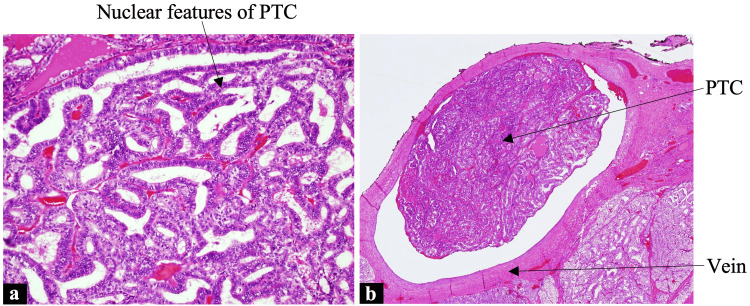
Histology (a) Thyroid specimen showing nuclear features of PTC (arrow) (hematoxylin-eosin stain; 4x magnification). (b) Venous invasion. (hematoxylin-eosin stain; 4x magnification). PTC, papillary thyroid carcinoma

He underwent total thyroidectomy and bilateral neck dissection. Histopathology showed a 5-cm solid pattern PTC in the right lobe of the thyroid gland with focal columnar cell features, extensive lymphovascular invasion including venous invasion, involvement of margins, and multiple lymph nodes with metastasis. The most significant focus of the tumor in the lymph node was 3.7 cm without extranodal extension. Based on the histopathology and the subsequent radiological evidence, his disease was classed as stage II and T3a(s)N1bM1 (tumor, node, metastasis) according to the 8th edition of the American Joint Committee on Cancer classification (Figure 1) [4].

Postoperatively, he developed euvolemic hyponatremia. At this stage, the surgical team involved endocrinologists. Upon further questioning, he gave a history suggestive of hypogonadism, headaches, and blurred vision. Biochemical evaluation, including a short Synacthen test, suggested panhypopituitarism and mild hyperprolactinemia (Table 1).

**Table 1:** Baseline biochemistry results ACTH, adrenocorticotropic hormone; FSH, follicular stimulating hormone; FT4, free thyroxine; LH, luteinizing hormone; Na, sodium; TSH, thyroid-stimulating hormone

Labs	Result	Reference range
TSH	0.008 mU/L	0.27-4.2 mU/L
FT4	10.4 pmol/L	12-22 pmol/L
Baseline ACTH (09:00 am)	4 ng/L	5-60 ng/L
Baseline cortisol (09:00 am)	136 nmol/L	240-620 nmol/L
30-minute serum cortisol level post-ACTH injection (09:30 am)	388 nmol/L	>450 nmol/L
60-minute serum cortisol level post-ACTH injection (10:00 am)	401.7 nmol/L	>550 nmol/L
Prolactin	55.4 ug/L	4.1-18.40 ug/L
Testosterone	<0.087 nmol/L	9.9-27.8 nmol/L
LH	<0.1 IU/L	1.7-8.6 IU/L
FSH	0.3 IU/L	1.5-12.4 IU/L
Serum Na level after starting cortisol replacement	151 mmol/L	135-147 mmol/L
Serum osmolality	295 mOsm/Kg	275-300 mOsm/Kg
Urine osmolality	188 mOsm/Kg	300-900 mOsm/Kg
Random urine Na	54 mmol/L	40-220 mmol/L

**Figure 2:**
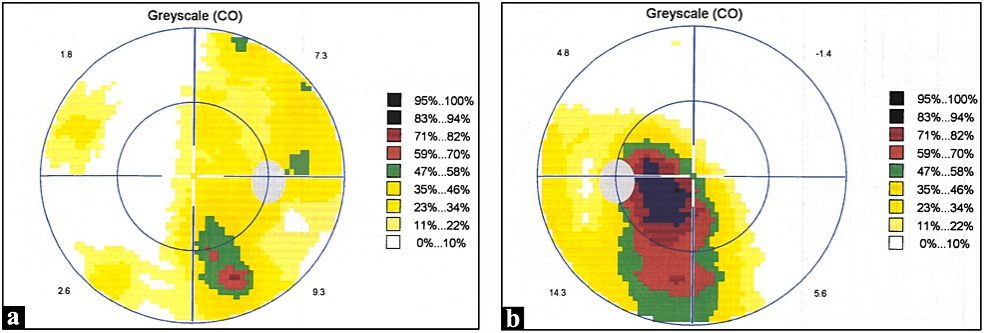
Humphrey’s visual field test results showing evidence of chiasmal compression (a) Patient's right eye. (b) Patient's left eye.

Visual field assessment confirmed bitemporal hemianopia (Figure 2). 

**Figure 3:**
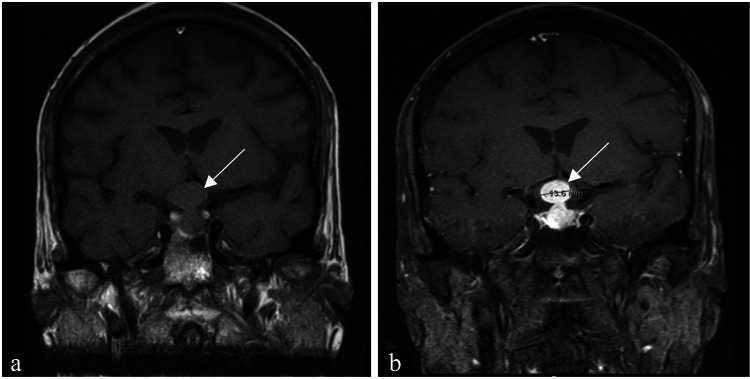
MRI scan (a) MRI scan of the pituitary gland showing an iso-intense dumbbell-shaped lesion (arrow) with thickening of the stalk. (b) Homogenous enhancement of the lesion (arrow) after contrast. MRI, magnetic resonance imaging

The pituitary gland's magnetic resonance imaging (MRI) showed a 1.3-cm pituitary lesion compressing the optic chiasm (Figure 3).

**Figure 4:**
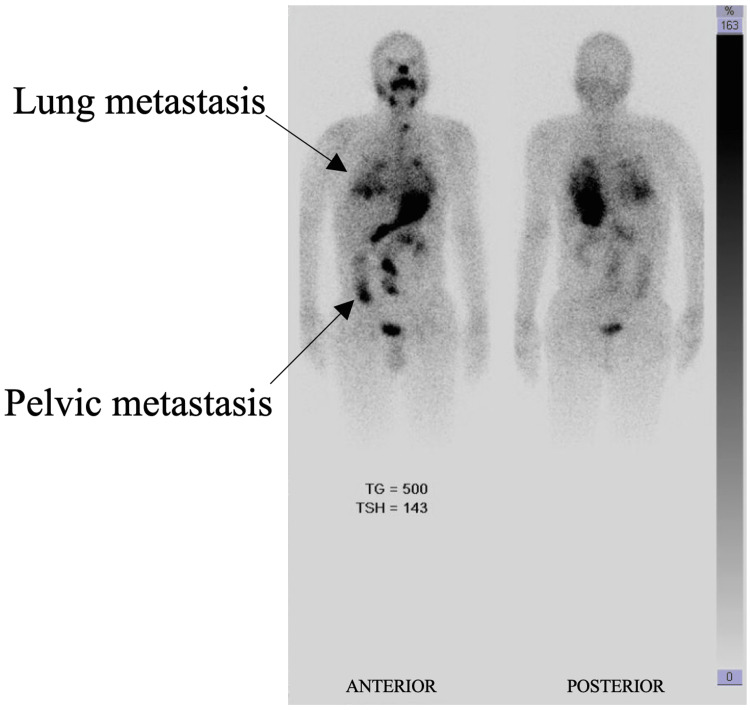
I-123 whole-body scan showing diffuse iodine avid bone and lung metastasis I-123, iodine-123; TG, thyroglobulin; TSH, thyroid-stimulating hormone

Iodine-123 (I-123) whole-body scan using recombinant TSH (rTSH) and steroid coverage showed diffuse skeletal (ribs, vertebra, pelvis) and lung uptake and no iodine uptake at the pituitary gland region (Figure 4).

**Figure 5:**
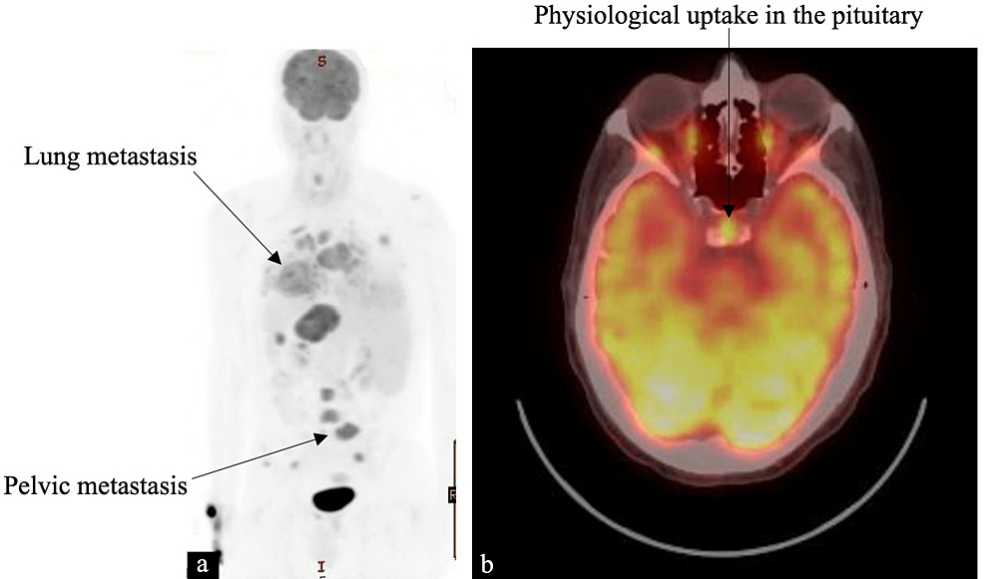
FDG-PET scan (a) Diffuse FDG avid disease in the bones and lungs. (b) Physiological FDG uptake in the pituitary gland (arrow). FDG, fluorodeoxyglucose; PET, positron emission tomography

We used steroids to minimize the risk of rapid expansion of the pituitary lesion. Fluorodeoxyglucose-positron emission tomography (FDG-PET) was positive for diffuse skeletal and lung metastasis and a physiological uptake in the pituitary region (Figure 5).

We initiated hormonal replacement, including glucocorticoids, followed by levothyroxine. After starting steroid replacement, the patient developed clinically and biochemically proven central DI treated with desmopressin (Table 1). The differential diagnosis at that stage included an atypical pituitary macro adenoma versus a metastatic lesion in the pituitary gland, given the aggressive disease behavior and the atypical presentation.

**Figure 6:**
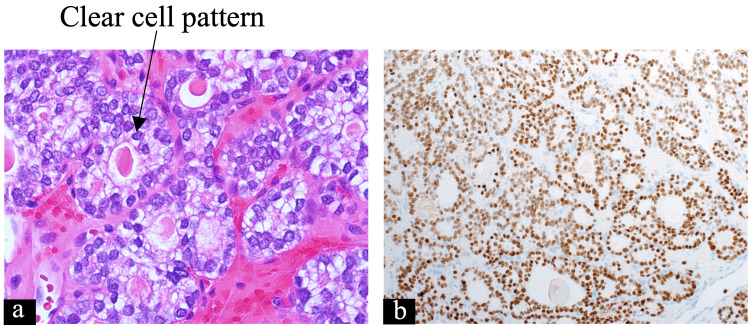
Pituitary pathology (a) Pituitary tissue showing clear cell pattern in metastatic PTC (arrow) (hematoxylin-eosin stain; 20x magnification). (b) TTF-1 stain of pituitary tissue that is positive for nuclear staining in brown (TTF-1 immunohistochemical stain; 4x magnification). PTC, papillary thyroid cancer; TTF-1, thyroid transcription factor-1

Due to visual compromise and optic nerve compression, he underwent an urgent trans-sphenoidal debulking surgery on the pituitary lesion. A bluish vascular tumor posterior to the normal gland was identified. The tumor was very vascular and friable, with abundant intra-operative bleeding. Adhesion of the tumor, attachment to the ophthalmic and internal carotid artery, and significant bleeding made any meaningful debulking impossible. The surgeons concluded the procedure with minor debulking and securing hemostasis. Pathology showed metastatic PTC with clear cell features (Figure 6). The visual symptoms did not improve, and the visual field assessment showed persistent bitemporal hemianopia. His postoperative pituitary MRI showed a progression in size with persistent optic nerve compression.

The critical location of the pituitary lesion and the rapid progression dictated the treatment course. The patient had been deemed a case of high surgical risk due to a highly vascular and friable tumor. The pituitary lesion was non-iodine avid (Figure 4), and thus we opted for external beam radiation therapy (EBRT), a more favorable option over radioactive iodine (RAI). He received 1,200 centigray (cGy) with Gamma knife radiosurgery with steroid cover.

In our case, the aggressive histological and clinical variant of PTC consisted of multiple metastatic sites involving large volume pulmonary, skeletal, and chest wall lesions coupled with crucial macro metastatic pituitary mass that had threatened vision with the possibility of life-threatening pituitary apoplexy. We offered him RAI to treat other iodine avid metastases in the lungs and bones and also offered EBRT to target skeletal lesions. We discussed using systemic treatment with tyrosine kinase inhibitor. Despite multiple meetings with the patient and counseling him about the disease course and the treatment options, he refused further treatment and did not return for further care.

## Discussion

PM occurs through the hematogenous spread, direct invasion from skull base metastasis, or meningeal spread through the suprasellar cistern [5]. The hematogenous route is not a typical route for dissemination of PTC, and tumors with a marked tendency toward hematogenous metastasis, such as follicular TC and PTC with extensive vascular invasion, have been reported to cause PM [6]. The lack of bony skull involvement, extensive vascular invasion, and diffuse systemic metastasis suggest that the hematogenous route was our case's predominant course of spread.

PM from TC can present in a very variable period. The presentation of PM can pre-date the diagnosis of TC. Barbaro et al. described one of their patients who presented with neurological manifestations from a sellar tumor [6]. The patient underwent surgical resection, and the pathology showed metastasis from the previously undiagnosed TC. The PM can present from a few months [6] to decades [7] after the diagnosis of TC and total thyroidectomy. In some cases, the diagnosis of TC and PM can present simultaneously, like in our case.

The manifestation of PM can be variable. Some patients present only with pressure symptoms such as optic nerve compression and cranial nerve deficit without the hormonal disorder [6], while some present just with hormonal insufficiency [8]. Our patient experienced both the pressure symptoms, such as bitemporal hemianopia and panhypopituitarism. Pituitary involvement was suspected early on in our case, given the inappropriately low TSH in the face of low FT4 levels, hyponatremia, and other symptoms of pituitary hormonal deficiency.

Radiological features are neither sensitive nor specific to differentiate an incidental pituitary adenoma from the PM. A dumbbell-shaped intrasellar and suprasellar tumor, like the one seen in our case, with a clear indentation at the level of the diaphragma sellae indicates the rapid growth of the lesion and, if present, is indicative of early stages of PM. This feature, in addition to the iso-intensity of the lesion on the T1-weighted image, thickened pituitary stalk, and homogenous enhancement after gadolinium contrast, supported PM diagnosis [1].

Our patient had central hypothyroidism due to PM. Therefore, we did not use the traditional method of levothyroxine withdrawal to achieve the required elevated TSH levels before performing the diagnostic I-123 whole-body scan. We used rTSH, which is not licensed for use in patients with metastatic PTC. However, the American Thyroid Association has endorsed its use in patients with pituitary disease (recommendation 75) [9].

In line with the ATA recommendations, we prioritized the treatment of central nervous system metastasis with surgery and radiotherapy before considering the usual therapeutic approach, which involves radioiodine ablation of the systemic metastasis [9].

## Conclusions

The pituitary gland is a rare site for metastasis in PTC that can present before, after, or simultaneously with the presentation of PTC. Symptoms of pituitary hormonal deficiency can easily be overlooked as these are nonspecific. Clinicians should exercise vigilance and a high index of suspicion for PM when a patient with any pre-existing cancer presents with visual disturbance, cranial nerve deficit, or symptoms suggestive of hormonal deficiency. We advocate that surgeons should always involve endocrinologists before performing any surgery on the endocrine organs to ascertain the integrity of the endocrine function of the glands.

## References

[1] Komninos J, Vlassopoulou V, Protopapa D, Korfias S, Kontogeorgos G, Sakas DE, Thalassinos NC (2004). Tumors metastatic to the pituitary gland: case report and literature review. J Clin Endocrinol Metab.

[2] Teears RJ, Silverman EM (1975). Clinicopathologic review of 88 cases of carcinoma metastatic to the putuitary gland. Cancer.

[3] Morita A, Meyer FB, Laws ER Jr (1998). Symptomatic pituitary metastases. J Neurosurg.

[4] Tuttle RM, Haugen B, Perrier ND (2017). Updated American Joint Committee on Cancer/Tumor-Node-Metastasis Staging System for Differentiated and Anaplastic Thyroid Cancer (Eighth Edition): what changed and why?. Thyroid.

[5] Fassett DR, Couldwell WT (2004). Metastases to the pituitary gland. Neurosurg Focus.

[6] Barbaro D, Desogus N, Boni G (2013). Pituitary metastasis of thyroid cancer. Endocrine.

[7] Bell CD, Kovacs K, Horvath E, Smythe H, Asa S (2001). Papillary carcinoma of thyroid metastatic to the pituitary gland. Arch Pathol Lab Med.

[8] Masiukiewicz US, Nakchbandi IA, Stewart AF, Inzucchi SE (1999). Papillary thyroid carcinoma metastatic to the pituitary gland. Thyroid.

[9] Haugen BR, Alexander EK, Bible KC (2016). 2015 American Thyroid Association Management Guidelines for Adult Patients with Thyroid Nodules and Differentiated Thyroid Cancer: The American Thyroid Association Guidelines Task Force on Thyroid Nodules and Differentiated Thyroid Cancer. Thyroid.

